# Insulin Receptor Substrate 1 Gly972Arg (rs1801278) Polymorphism Is Associated with Obesity and Insulin Resistance in Kashmiri Women with Polycystic Ovary Syndrome

**DOI:** 10.3390/genes13081463

**Published:** 2022-08-17

**Authors:** Shayaq Ul Abeer Rasool, Mudasar Nabi, Sairish Ashraf, Shajrul Amin

**Affiliations:** 1 Department of Biotechnology, University of Kashmir, Srinagar 190006, India; 2 Department of Biochemistry, University of Kashmir, Srinagar 190006, India

**Keywords:** polycystic ovary syndrome, insulin resistance, IRS-1, Gly972Arg, obesity

## Abstract

Background: Polycystic ovary syndrome (PCOS) is commonly associated with metabolic abnormalities such as hyperinsulinemia, insulin resistance and obesity. The genetic variants of genes regulating insulin action, expression and regulation are suggested as possible factors involved in development and severity of clinical manifestations in PCOS. Aim: We investigated whether IRS-1Gly972Arg (rs1801278) polymorphism is associated with increased risk of PCOS in Kashmiri women. The correlation of various clinical, metabolic and hormonal markers with rs1801278 single nucleotide polymorphism was analyzed. The genotypic–phenotypic association of clinical manifestations of PCOS with the tested genetic variant was also assessed. Results: There were no significant differences in allele frequency (OR = 0.87, CI = 0.59–1.29, χ^2^ = 0.456, *p* = 0.499) or genotypic distribution (χ^2^ = 3.73, *p* = 0.15) between PCOS women and controls. No significant association was also found in the dominant (OR = 1.63, χ^2^ = 0.377, *p* = 0.53), recessive (OR = 0.79, χ^2^ = 1.01, *p* = 0.31) or heterozygote vs. homozygote (OR = 1.34, χ^2^ = 1.53, *p* = 0.22) genotype model analysis. The genotype–phenotype correlation analysis showed that the Arg allele was significantly associated with increased central adiposity markers hip circumference (*p* = 0.012), and body adiposity index BAI (*p* = 0.002) in the recessive model in PCOS women. The two-hour glucose (*p* = 0.04) and insulin resistance marker HOMA (*p* = 0.44) were significantly higher in Arg allele carriers. The androgen excess markers dehydroepiandrosterone sulfate DHEAS (*p* = 0.02), Ferriman–Gallwey score (*p* = 0.012), prevalence of acne, alopecia and hirsutism (all *p* < 0.01) were significantly elevated in the wild-type GG genotype. Conclusions:IRS-1Gly972Arg genetic variant does not increase the risk of PCOS in Kashmiri women. However, this polymorphism is associated with clinical manifestations of insulin resistance, obesity and hyperandrogenism, suggesting its possible role in variable phenotypic manifestations of PCOS.

## 1. Introduction

Polycystic ovary syndrome (PCOS) is a complex, multifactorial endocrine–metabolic disorder. Insulin resistance and obesity, present in ~40–80% women with PCOS [1,2], are considered basic denominators of metabolic and reproductive complications such as diabetes, cardiovascular disorders and infertility in such women [3,4]. The etiology of PCOS is polygenic. The androgen biosynthesis, gonadotropin, insulin synthesis and regulation genes are suggested to play an essential role in pathogenesis and progression [5,6,7]. Genetic variants such as single nucleotide polymorphisms (SNPs) are extensively investigated as potential players that can explain the phenotypic manifestations of complex disorders including PCOS [8].

Insulin receptor substrate 1 (IRS-1) gene is 68.4 kb in length containing two exons and is located at 2q36. It encodes a 131.6 kDa protein which is an important player in the insulin signaling pathway. The insulin-induced phosphorylation of tyrosine residues of β-subunit of insulin receptor (INSR) induces downstream activation via phosphorylation in cytosolic IRS proteins by INSR [9]. Besides tyrosine phosphorylation, IRS-1 is phosphorylated at multiple serine/threonine residues. Activated IRS-1 acts as a docking and activation site for various proteins such as growth factor receptor-boundprotein2 (Grb2/SOS complex),non-catalytic region of tyrosine kinase (NcK) and p85 subunit of phosphatidylinositol 3-kinase (PI3K) [10]. This leads to the activation of PI3K and mitogen activated protein kinase (MAPK) pathways that initiate the physiological effects of insulin. The phosphorylation is dependent upon the affinity and specificity of phosphorylation sites within IRS-1 with tyrosine kinase [11,12]. Therefore, alterations at these sites can alter the insulin signal transduction. The SNP present in exon 1 at codon 972 (rs1801278) results in the substitution of arginine in place of glycine in IRS-1 protein. This substitution has effects on the tertiary structure of IRS-1 and results in disturbed downstream signaling and finally can lead to impaired glucose tolerance and insulin resistance [13,14]. The polymorphism decreases the binding and activation of the p85 subunit of PI3K, Grb2, AKT andeNOS with IRS-1 resulting in compromised response to insulin stimulation. The Gly972Arg amino acid substitution has been found to be associated with insulin resistance, type 2 diabetes and cancer [13,15,16].

In PCOS, though positive associations have been reported for this polymorphism in some Caucasian and Asian studies, other studies have reported lack of association of this SNP with PCOS [17,18,19,20,21]. Although this genetic variant has a strong role in drug response, the impact of IRS-1 Gly972Arg polymorphism on different clinical manifestations of characteristic features of PCOS such as hyperandrogenism and chronic anovulation has not been thoroughly investigated. Since various genetic variants show variable effects in different ethnic and geographical settings, it is therefore essential to investigate the association of this single nucleotide polymorphism in different ethnic populations to understand the risk this SNP imparts on the women of different ethnicities affected by PCOS. In this study, we investigated the effect of the IRS-1 Gly972Arg variant on various key metabolic, reproductive and cardiovascular markers in North Indian Kashmiri women with PCOS. The study also investigates the role of this polymorphism in the clinical manifestations of hyperandrogenism, insulin resistance and hormonal dysfunction.

## 2. Material and Methods

### 2.1. Recruitment of Subjects

This case-control study included 349 women (ages 16–30). The women visiting the endocrinology outpatient clinic of Sher e Kashmir Institute of Medical Sciences (SKIMS), India, for PCOS-related symptoms such as hirsutism, acne, obesity, infertility or menstrual irregularities as main complaints from June 2015 to March 2018 were evaluated for PCOS. Out of 700 women evaluated, 249 women were diagnosed with PCOS and were recruited for the study. The diagnosis of PCOS was carried out according to the revised 2003 Rotterdam criteria [22]. The participants were screened to exclude hyperprolactinemia, thyroid dysfunction, Cushing’s syndrome, congenital adrenal hyperplasia and androgen-secreting ovarian/adrenal tumors. The control group consisted of 100 age-matched healthy volunteers with regular menstrual cycles and no clinical signs of hyperandrogenism on physical examination. All subjects were ethnic Kashmiris/North Indians living in the Kashmir province of India and had not received hormonal therapy for at least 3 months before hormonal assays [7]. The sample size was calculated according to Hong and Park’s criteria for genetic association studies [23].

### 2.2. Ethics Statement

This study was approved by the Sher-i-Kashmir Institute of Medical Sciences Srinagar institutional ethics committee under IEC approval no. SIMS 1-31/IEC-SKIMS/2013/6592. Subjects were recruited after written informed consent was obtained from them.

### 2.3. Anthropometric and Clinical Evaluation

Detailed clinical history including menstrual history, acne, alopecia and acanthosis nigricans was taken from the participants. The general anthropometric variables weight, height, systolic and diastolic blood pressure, body mass index (BMI: weight [kg]/height [m^2^]), waist–hip ratio (WHR) and hirsutism were recorded. The hirsutism was measured visually using the Ferriman–Gallwey scoring system in which nine androgen sensitivity body parts were examined for hair growth. Each body part was scored from 0 to 4, and a cumulative score of ≥8 was considered significant. Height was measured in a standing position without shoes using a height measuring scale. Weight was measured using a Krups weighing scale with light clothing and without shoes. For determination of WHR, waist circumference was measured in a standing position as the minimum value between the iliac crest and the lateral costal margin at the end of a gentle expiration, and hip circumference was calculated as the maximum value over the buttocks. Blood pressure (BP) was measured in a relaxed sitting position after 5–10 min rest using a Diamond Mercurial Sphygmomanometer Blood Pressure Monitor.

### 2.4. Biochemical and Hormonal Assessment

The blood samples were obtained from the participants on days 2–3 or the early follicular phase of the menstrual cycle or withdrawal bleeding with progesterone for subjects with amenorrhea. The blood was placed in clot activator vials for obtaining serum and Na_2_EDTA vials for DNA isolation. The serum was separated by centrifugation at 3000 rpm for 10 min at 4 °C within 2 h of blood collection. The serum concentration of luteinizing hormone (LH), follicle-stimulating hormone (FSH), total testosterone (TT), thyroid-stimulating hormone (TSH) and prolactin (PRL) were measured with a radioimmunoassay RIA kits (Immunotech, Prague, Czech Republic) on a Beckman Coulter UniCelDxl 800 (Access Immunoassay system, Brea, CA, USA) [7]. Enzyme-linked immunosorbent assays were used to measure sex hormone-binding globulin (SHBG Kit, DGR Instruments GmbH, Marburg, Germany), androstenedione, dehydroepiandrosterone sulfate (DHEAS) and fasting insulin using ELISA kits (Calbiotech, CA, USA).

The free androgen index (FAI) was derived using the formula

$$\mathrm{FAI}=\frac{\mathrm{TotalTestosterone}\;\;\;\mathrm{nmol}/L}{\mathrm{SHBG}\;\mathrm{nmol}/L}\times 100$$

The glucose and lipid indices were measured in study subjects. The glucose and insulin were measured in fasting state after a 12 h overnight fast and two-hour glucose post 75 g oral glucose tolerance test (OGTT). The level of glucose was measured with by Accu-Chek active blood glucose monitoring system (Roche Diagnostics, Mannheim, Germany). 

Insulin resistance was estimated using the homeostatic model assessment of insulin resistance (HOMA) method derived using the formula [24]:

HOMA=Fasting glucose (mg/dL)×Fasting Insulin (μIU/mL)/405

The insulin sensitivity was estimated with the quantitative insulin sensitivity check index (QUICKI) according to the formula:

QUICKI=1/log(FastingInsulinu U/mL)+log(fasting glucosem g/dL)

The lipid accumulation product (LAP) was calculated using the formula:

LAP=(waistcircumfrence−58)×Triglycerides

The body adipose index (BAI) was calculated using formula of Bergman et al. [25]

$$\mathrm{BAI}=\frac{\mathrm{hipcircumfrence}\;\left(\mathrm{cm}\right)}{{\mathrm{height}}^{1.5}}-18$$

The biochemical parameters such as fasting serum lipid profile (cholesterol (CHOL) and triglycerides (TG), urea, uric acid, creatinine, alanine aminotransferase (ALT) and aspartate aminotransferase (AST) were determined with enzymatic methods using Erba bioassay diagnostic kits ERBA Diagnostics Manheim, Mumbai, India) and analyzed on Chem7 biochemistry analyzer (Transasia, Mumbai, India).

### 2.5. Genotyping

The genomic DNA was isolated from peripheral blood leukocytes by using QIAamp DNA mini kit (QIAGEN, Hilden, Germany). The DNA concentration was measured at OD260 and purity was checked by the ratio OD260/OD280 ratio using a nanodrop 2000c spectrophotometer (Thermo Fisher Scientific, Wilmington, NC, USA) and integrity checked by subjecting DNA to electrophoresis on 1% agarose gel. For genotyping, IRS-1 gly972Arg polymorphism was analyzed with polymerase chain reaction restriction fragment length polymorphism (PCR–RFLP) The PCR amplification was carried out using primers described by Pappalardo et al. [20]. The forward 5′-GCTTTCCACAGCTCACCTTC-3′ and reverse 5′-GGTAGGCCTGCAAATGCTA-3′ primers were used. PCR reaction conditions consisted of an initial denaturation at 94 °C for 5 min followed by 35 cycles, each cycle with denaturation at 94 °C for 30 s, annealing at 52 °C for 40 s and extension at 72 °C for 40 s and a final extension at 72 °C for 7 min using an AgilentSurecycler8800 (Agilent, Santa Clara, CA, USA). The PCR product was subjected to restriction fragment length polymorphism (RFLP) and digested by 10 U of SmaI restriction enzyme (New England Biolabs, Wilmington, NC, USA) at 25 °C. The digested products were separated on 2.5% agarose gel and visualized on an Odyssey FC imaging system (LI-COR Biosciences, Lincoln, Nebraska, USA. The G and A alleles could be distinguished as bands of 198 and 171 plus 27 bp, respectively (Figures S1 and S2).

### 2.6. Statistical Analysis

All continuous variables are presented as mean ± standard deviation and categorical variables as numbers and percentages. Clinical, anthropometric, hormonal and metabolic variables were compared between PCOS and controls and genotype groups using the unpaired student t-test, and non-parametric variables such as the prevalence of clinical symptoms were compared with the chi-squared test. The Pearson chi-squared (χ^2^) test was used to reveal differences in allele and genotype frequencies and test deviations of genotype distribution from the Hardy–Weinberg equilibrium between PCOS and controls. Odds ratios and 95% confidence intervals were calculated to test the relative risk of dominant, recessive and additive models. One-way analysis of variance (ANOVA) independent standard weighted-means analysis was used to compare multiple groups in an additive genotype model followed with a post hoc Bonferroni test for intergroup association. The statistical analysis was performed using the statistical computation website Vassar Stats (http://vassarstats.net/ accessed from January 2020 to January 2022). A *p* value of <0.05 was considered statistically significant.

## 3. Results

The baseline anthropometric, biochemical and hormonal parameters in PCOS (*n* = 249) and controls (*n* = 100) showed that women with PCOS have significantly higher hormonal, biochemical and metabolic characteristics than controls.

We found the allelic frequency in PCOS women for the G and A alleles was 79.31% and 20.68%, respectively, whereas allelic frequency in controls was 77% and 23% for G and A alleles respectively. There was no significant association in allele frequency between cases and controls (OR = 0.87, CI = 0.59–1.29, χ^2^ = 0.456, *p* = 0.49). The wild GG genotype was present in 61.84% of PCOS cases and 56% of controls, while the GA genotype was present in 34.93% of cases and 42% of controls. The AA genotype was found in 3.21% of cases and 2% of controls. There was no significant difference between these genotypes between cases and controls (χ^2^ = 3.73, *p* = 0.15). The Hardy–Weinberg equilibrium in PCOS (χ^2^ = 1.05, *p* = 0.306), controls (χ^2^ = 3.45, *p* = 0.06) and overall χ^2^ = 3.54, *p* = 0.06 for this polymorphism. The results are given in Table 1

The genotype frequencies were compared in dominant (GG + GA vs. AA), recessive (GG vs. GA + AA) and heterozygote vs. homozygote (GA vs. GG + AA) genotype models. We found no significant association in dominant (OR = 1.63, χ^2^ = 0.377, *p* = 0.539), recessive (OR = 0.79, χ^2^ = 1.01, *p* = 0.313) or heterozygote vs. homozygote (OR = 1.34, χ^2^ = 1.53, *p* = 0.22) models (Table 2).

### Genotypic–Phenotypic Association Analysis of IRS-1 Gly972Arg (rs1801278) SNP

To understand the effect of IRS-1 (G/A) polymorphism on clinical, hormonal, metabolic and biochemical parameters in PCOS cases and control women, the data were analyzed for recessive (GG vs. GA + AA), dominant (GG + GA vs. AA) and additive (GG vs. GA vs. AA) genotype models.

**Figure 1:**
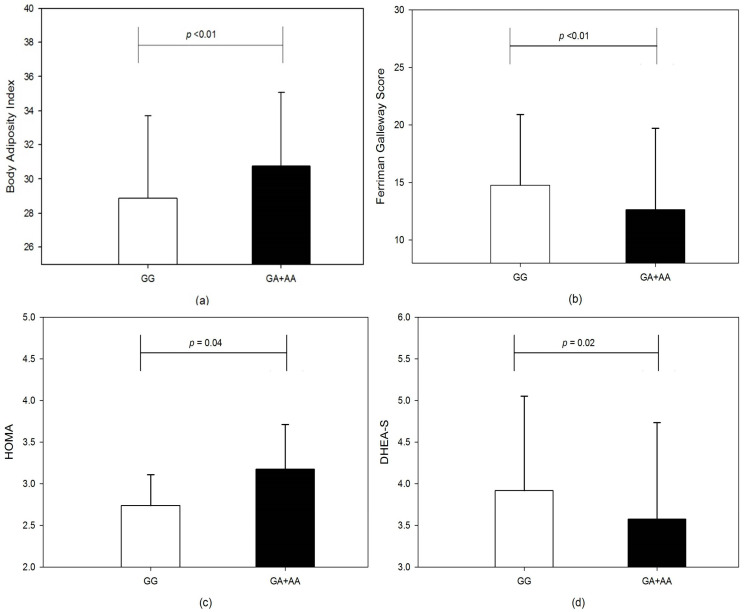
The bar graphs show a comparison between GG and GA + AA genotypes for (**a**) body adiposity index, (**b**) Ferriman Gallwey score, (**c**) HOMA and (**d**) DHEA-S, in the recessive genotype model.

The comparison of various anthropometric, clinical, biochemical and hormonal parameters between PCOS and healthy controls is summarized in Table 3. In the recessive model, the IRS 1 Gly972Arg polymorphism showed significant differences when phenotypic features of genotypes were compared. Although the markers for hyperandrogenism such as free androgen index (FAI) and total testosterone were comparable (*p* > 0.05), the DHEA-S level was significantly higher (3.92 ± 1.13 vs. 3.57 ± 1.16, *p* = 0.02) in GG genotype than the GA + AA genotypes (Figure 1d) The mean FG score for hirsutism was significantly higher (*p* = 0.012) in the wild-type GG 14.77 ± 6.15 as compared to GA + AA 2.63 ± 7.08 (Figure 1b). The BMI was marginally higher in Arg allele carriers (23.87 ± 4.41 vs. 24.96 ± 4.76; *p* = 0.067). Similarly body adiposity index was significantly higher (*p* < 0.01) in variant genotype (Figure 1a) but WHR (0.89 ± 0.09 vs. 0.88 ± 0.07, *p* = 0.356) was comparable between GG vs. GA + AA genotypes. The hip circumference (92.44 ± 8.25 vs. 95.15 ± 8.19; *p* = 0.012) was elevated in GA + AA genotypes than GG genotypes. The fasting insulin level for GG vs. GA + AA genotypes, 12.86 ± 6.66 vs. 14.61 ± 7.22, *p* = 0.052, was also marginally significant between the two groups. Though the fasting glucose level was comparable (*p* > 0.05) between the two groups, there was a significant increase in 2 h glucose levels 117.69 ± 19.11 vs. 112.82 ± 17.21, *p* = 0.044). Insulin sensitivity marker, QUICKI, was lower in GA + AA compared to the GG group (0.326 ± 0.02 vs. 0.331 ± 0.02, *p* = 0.056), and insulin resistance marker HOMA was elevated (2.74 ± 1.55 vs. 3.17 ± 1.75; *p* = 0.04) in variant allele carrier GA + AA genotype (Figure 1). There was a significant association for urea (23.45 ± 6.20 vs. 21.61 ± 5.43, *p* = 0.018) and AST (32.81 ± 13.78 vs. 29.10 ± 9.60, *p* = 0.02). In controls, although other metabolic, hormonal and biochemical parameters were comparable in the recessive genotype model, the weight was significantly higher in the GA + AA group than in the GG group (50.77 ± 6.43 vs. 53.43 ± 6.86; *p* = 0.048). The comparison of biochemical, hormonal and metabolic parameters in the GG vs. GA + AA genotype model is summarized in Table 4.

In the dominant genotype model, we found no significant association between any tested clinical, biochemical or hormonal parameters in cases. In controls, we found testosterone (33.68 ± 15.29 vs. 56.77 ± 19.29; *p* = 0.037), FAI (2.15 ± 1.55 vs. 5.21 ± 4.39; *p* = 0.008), FSH/LH (1.00 ± 0.45 vs. 2.72 ± 1.92; *p* = 0.001) and urea (21.21 ± 3.32 vs. 26.62 ± 8.09; *p* = 0.028) were significantly increased in the AA genotype than GG + GA genotypes (Table S1). In GG vs. GA vs. AA, we found significant difference between BAI (28.86 ± 4.84 vs. 30.74 ± 4.17 vs. 30.89 ± 5.92; *p*= 0.002), urea (23.45 ± 6.20 vs. 21.31 ± 5.44 vs. 24.88 ± 4.39; *p* = 0.016) and AST (32.81 ± 13.78 vs. 28.60 ± 8.89 vs. 34.63 ± 15.10; *p* = 0.031) for GG vs. GA vs. AA genotypes. We found no significant association between any tested clinical, biochemical or metabolic parameters in the additive genotype model for controls (Table S2).

The phenotypic analysis for the Gly/Gly genotype showed a significantly higher proportion of PCOS women presented with clinical signs of hyperandrogenism. The proportion of PCOS women with acne, alopecia and hirsutism was higher in the wild-type GG genotype than in GA, AA or both genotypes combined. The number of women with menstrual disturbances was also significantly higher (*p* < 0.001) in the GG genotype compared to other genotypes. In contrast to significantly higher mean values of HOMA in GG genotype (Figure 1c), the proportion of women with markers of insulin resistance, acanthosis nigricans and HOMA > 2.71 was comparable between genotypes. There was also no significant difference (*p* > 0.05) in the overall percentage of women with obesity (BMI > 25 kg/m^2^) in the genotypes compared (Table 5).

## 4. Discussion

Insulin resistance, a common finding in women with PCOS, plays an important role in metabolic and endocrine dysfunction in affected women [1,26]. The defects in the insulin signaling pathway are important targets for understanding the pathophysiology of this complex multifactorial syndrome. Insulin receptor substrate-1 (IRS-1) is a critical element in insulin signaling pathways, and mutations in theIRS-1gene have been reported to have a role in determining susceptibility to traits such as impaired sensitivity to insulin-related to type 2 diabetes [15,27]. This polymorphism assumes clinical significance as it has been shown to affect the response of insulin sensitizers and oral antidiabetic drugs such as metformin in PCOS and type 2 diabetes [28]. In the present study, we did not find a significant association between IRS-1gene Gly972Arg polymorphism and PCOS. The allele frequency was not significantly different between controls or PCOS cases (OR = 0.87, CI = 0.59–1.29, χ^2^ = 0.456, *p* = 0.49). We found the frequency of the G and A allele was 79.3% and 20.7% in cases and 77% and 23% in controls, which is consistent with the allele frequency reported in Caucasian/Greek (82%) [29], Chilean (84.0%) [16], French (88.7%) [14] and Slovakian (88.7%) [14] PCOS women. Our results are consistent with other Asian studies, specifically Indian (*p* = 0.493) [30], Korean (*p* > 0.05) [31], Taiwanese (*p* = 0.66) [32] and Japanese populations (*p* = 0.109) [33]. No significant association of this genetic variant was reported in Caucasian populations, namely Spanish (*p* = 0.74) [34], American (*p* = 0.75) [35], Croatian (*p* = 0.14) [36] and Dutch (*p* = 0.183) [37] case control studies. However, contrary to our results, Christopoulos et al. reported a positive association in Greek women with PCOS (*p* < 0.05) [38]. In South Indian women, Thangavelu et al. investigated 169 PCOS cases and reported a significant association (*p* < 0.001) between IRS-1 Gly972Arg SNP and PCOS [18]. In a meta-analysis, Shi et al. analyzed 16 IRS-1 Gly972 polymorphism and PCOS case control studies including 1851 PCOS cases and 2017 controls and concluded that there was a significant association (*p* = 0.004, OR = 0.57, 95% CI: 0.39–0.84) of the polymorphism with PCOS [21]. Table 6 summarizes the results of previous association studies of IRS-1 Gly972Arg polymorphism and compares them with the present study.

Further, the genotype–phenotype effect analysis of the clinical, hormonal, metabolic and biochemical parameters showed that in the recessive model, the mean FG score was significantly higher (*p* = 0.01) in the GG genotype than GA + AA genotype. Although testosterone and FAI values were higher in the wild-type, the differences were not statistically significant. The significantly higher mean FG score and hirsutism can be explained by the significantly higher DHEAS level (*p* = 0.02) in GG genotypes. Pappalardo et al. also reported significantly higher mean FG score in GG genotypes in PCOS women of Italian origin [41]. Villuendas et al. compared 100 PCOS and 48 healthy Spanish women for IRS-1 polymorphism and found the GG genotype had higher levels of androgens such as testosterone and androstenedione [34]. A study investigating the impact of IRS-1Gly972Arg on PCOS women of Greek origin also reported higher levels of testosterone, DHEAS and FAI in GG genotype [38].

We found significantly higher hip circumference (*p* = 0.012) and BAI (*p* = 0.002) in the A allele carrying the genotype combination (GA + AA). Christopoulos et al. also reported GA + AA genotypes with higher BMI and WHR, but the differences reported were not statistically significant [38]. A previous study in Spanish PCOS women also reported significantly higher BMI in GA genotypes than in controls [34]. In the wild-type GG genotype, two-hour glucose was significantly higher than in GA + AA genotypes. This finding is consistent with the findings of Villuendas et al. and Pappalardo et al., who also reported a significantly lower level of two-hour glucose level in the GA + AA genotype than in controls [34,41]. We found insulin levels were higher in GA + AA than in controls. Previous studies have found significantly higher insulin levels in A allele carriers, but our results were marginally significant. We also found urea levels were significantly altered in the presence of this polymorphism. Thameen et al. investigated 700 Mexican-American individuals and reported Gly972Arg polymorphism was associated with variations in glomerular filtration rate [43]. We found the insulin resistance marker, HOMA, was significantly higher (*p* = 0.04) in dominant GA + AA as compared to the GG genotype. We found insulin sensitivity was compromised in the GA + AA group as compared to the GG genotype which is consistent with the higher level of fasting insulin concentration found in this group. In the variant AA genotype, the increase in fasting insulin was marginally significant (*p* = 0.052), and insulin sensitivity was also marginally reduced (*p* = 0.056). Villuandes et al. also reported significantly higher HOMA (*p* = 0.009) in the variant allele containing genotypes [34]. Hence our results suggest IRS-1 Gly792Arg polymorphism may exert its effect on the phenotypic expression in PCOS by having protective functions in case of hirsutism or may lead to an increase in the risk of adiposity and insulin resistance. This is in accordance with previous studies that show IRS-1 G972R polymorphism is associated with failure of oral antidiabetic drugs in patients with type 2diabetes [44]. Insulin resistance in itself is a multifactorial disorder and requires the simultaneous presence of several genetic and epigenetic alterations. We did not observe a significant increase in the percentage of women with insulin resistance in Arg allele carriers; this suggests that this polymorphism does not significantly increase the risk of insulin resistance but can exacerbate this condition. The association testing in 4279 cases and 3532 controls conducted by Florez et al. did not find an association (OR 0.96, *p* = 0.60) of IRS-1 Gly972Arg polymorphism with type 2 diabetes and insulin resistance [45]. It has been found that IRS-1 G972R polymorphism is directly associated with the insulin receptors and inhibits autophosphorylation of INSR. This association has been found to occur in the β subunit of INSR that includes the tyrosine kinase domain at residues 966 and 1271 [46]. This interaction also involves INSR His1058 residue, indicating a relationship between INSR C/T polymorphism and IRS-1 Gly972Arg polymorphism. McGettric et al. demonstrated that IRS-1 Gly972Arg polymorphism inhibits phosphorylation of IGF-1R, having a similar effect to insulin signaling because the tyrosine kinase domains of both receptors are highly homologous [10]. It is also reported that IRS-1 polymorphism may interfere at the ATP-binding site at Lys1018 residue which may lead to a severe loss of tyrosine kinase activity. Thus, the association of G972R polymorphism with INSR and a decrease in the autophosphorylation of INSR can lead to a decrease in downstream signaling via the PI 3-kinase pathway.

There are some limitations to our study. Although our study included the highest number of cases and controls compared to previous studies conducted on analyzing INSR gene exon 17 C/T SNP (rs1799817) in PCOS, we could not analyze the required number of controls to establish a significantly powered study. Another limitation could be that the cases were recruited from a single tertiary healthcare institution which may not represent an unselected population and may lead to a selection bias.

## 5. Conclusions

This study shows for first time that the IRS-1 Gly972Arg polymorphism is not associated with increased risk of PCOS in North Indian/Kashmiri women but plays a role in phenotypic manifestations of this syndrome. The body adiposity index and insulin resistance marker HOMA value significantly increased in the presence of the variant allele. This shows that Arg substitution may increase the risk of central adiposity and insulin resistance in Kashmiri women with PCOS. However, this allele also imparts a protective role in presentation of clinical symptoms of hyperandrogenism and chronic oligo-anovulation. This indicates that this genetic variant may be involved in the variable presentation of endocrine–metabolic anomalies in women with PCOS.

## Figures and Tables

**Table 1:** Allele frequency, genotype distribution and Hardy–Weinberg equilibrium of IRS-1 Gly972Arg polymorphism in PCOS as compared with control women.

Allele/	Cases	Controls	Total	OR	χ^2^
Genotype	(*n* = 249)	(*n* = 100)	(*n* = 349)	(95% CI)	(*p*)
**G**	395 (79.3%)	154 (77.0%)	498	0.87 (0.59–1.29)	0.45 (0.499)
**A**	103 (20.7%)	46 (23.0%)	200		
**GG**	154 (61.85%)	56 (56.00%)	210 (63.90%)	-	3.73 (0.15)
**GA**	87 (34.94%)	42 (42.00%)	129 (27.22%)		
**GG**	8 (3.21%)	2 (2.00%)	10 (8.88%)		
**HWE**	1.05 (0.306)	3.45 (0.063)	3.54 (0.06)	-	-

PCOS: polycystic ovary syndrome, G and A: alleles for Gly972Arg polymorphism, CI: confidence interval, χ^2^: Pearson’s chi-squared test with Yates correction. Data of alleles are presented as number (%) of PCOS and controls. GG, GA and AA: genotypes of IRS-1 (G/A) polymorphism in PCOS and controls, HWE: χ^2^ Hardy–Weinberg equilibrium. (*p*): *p* value data of alleles are presented as number (%) of PCOS and controls.

**Table 2:** Genetic association models (dominant, recessive and homozygote vs. heterozygote) of IRS-1 Gly972Arg polymorphism in PCOS and controls.

Comparison	PCOS N/*n* (%)	Control N/*n* (%)	Odds Ratio (95% CI)	χ^2^	*p* Value
**GG + GA vs. AA**	241/8 (96.78% vs. 3.21%)	98/2 98% vs. 2%)	1.63 (0.34–7.80)	0.37	0.53
**GG vs. GA + AA**	154/95 (61.84% vs. 31.15%)	56/44 (56% vs. 44%)	0.79 (0.49–1.26)	1.01	0.31
**GA vs. GG + AA**	87/162 (34.93% vs. 65.06%)	42/58 (42% vs. 58%)	1.34 (0.83–2.16)	1.53	0.22

N/*n*: numbers in genotype compared, CI: confidence interval. χ^2^: Pearson chi-squared test.

**Table 3:** Comparison of anthropometric, hormonal, metabolic and biochemical parameters in PCOS cases and healthy controls.

Parameter	PCOS (*n* = 249)	Controls (*n* = 100)	*p* Value
Age (years)	22.43 ± 4.14	22.01 ± 3.17	0.36
Weight (kg)	59.83 ± 11.59	51.94 ± 6.72	<0.001 *
Height (m)	1.57 ± 0.05	1.57 ± 0.05	0.51
BMI (kg/m^2^)	24.28 ± 4.69	21.16 ± 2.48	<0.001 *
BAI	29.58 ± 4.73	28.62 ± 3.04	0.061
Waist (cm)	83.10 ± 11.13	77.01 ± 7.11	<0.001 *
Hip (cm)	93.47 ± 8.24	91.30 ± 6.33	0.018 *
WHR	0.89 ± 0.08	0.84 ± 0.06	<0.001 *
SBP (mmHg)	120.58 ± 7.51	118.96 ± 5.40	0.05
DBP (mmHg)	80.54 ± 5.89	79.32 ± 5.23	0.072
Menarche (years)	13.14 ± 1.14	13.28 ± 1.07	0.29
FG score	13.95 ± 6.58	4.53 ± 1.84	<0.001 *
LH (IU/L)	11.29 ± 9.54	6.65 ± 2.35	<0.001 *
FSH (IU/L)	6.09 ± 1.87	6.90 ± 1.97	<0.001 *
TT (ng/dL)	61.04 ± 23.93	34.15 ± 15.60	<0.001 *
PRL (ng/mL)	13.29 ± 5.61	10.42 ± 4.93	<0.001 *
TSH (μIU/L)	3.18 ± 1.44	2.99 ± 1.47	0.268
SHBG (nmol/L)	50.07 ± 21.67	64.91 ± 25.42	<0.001 *
Andro (ng/mL)	3.26 ± 0.86	2.26 ± 0.69	<0.001 *
DHEAS (ng/mL)	3.79 ± 1.15	2.9 ± 1.3	<0.001 *
Insulin F (μIU/mL)	13.53 ± 7.19	7.72 ± 5.39	<0.001 *
Glu F (mg/dL)	85.70 ± 8.59	84.54 ± 8.87	0.20
Glu 2 h (mg/dL)	115.47 ± 18.01	108.55 ± 14.29	<0.001 *
Chol (mg/dL)	154.73 ± 34.64	135.11 ± 19.30	<0.001 *
TG (mg/dL)	120.59 ± 35.57	102.73 ± 14.78	<0.001 *
HOMA IR	2.83 ± 1.68	1.62 ± 1.36	<0.001 *
QUICKI	0.338 ± 0.02	0.38 ± 0.05	<0.001 *
FAI	5.77 ± 5.62	2.21 ± 1.66	<0.001 *
LH/FSH	1.91 ± 1.27	1.04 ± 0.54	<0.001 *
LAP	35.16 ± 21.81	22.20 ± 9.08	<0.001 *
Urea (mg/dL)	22.76 ± 5.97	21.32 ± 3.47	0.024 *
UA (mg/dL)	4.27 ± 1.09	3.84 ± 0.75	<0.001 *
Creatinine (mg/dL)	1.03 ± 0.43	0.80 ± 0.13	<0.001 *
AST (U/L)	31.39 ± 12.46	18.30 ± 7.84	<0.001 *
ALT (U/L)	27.84 ± 13.82	23.46 ± 6.78	0.002 *

Data presented as mean ± SD, * *p* value < 0.05 was considered significant calculated by Student’s *t*-test. y: years, BMI: body mass index, WHR: waist–hip ratio, WHtR: waist to height ratio, FG score: Ferriman–Gallwey score, SBP: systolic blood pressure, DBP: diastolic blood pressure, LH: luteinizing hormone, FSH: follicle-stimulating hormone, TSH: thyroid-stimulating hormone, SHBG: sex hormone-binding globulin, DHEAS: dihydroepiandrosterone sulphate, FAI: free androgen index, BAI: body adiposity index, LAP: lipid accumulation product, AST: aspartate aminotransferase, ALT: alanine aminotransferase.

**Table 4:** Comparison of anthropometric, hormonal, metabolic and biochemical parameters of PCOS cases and healthy controls in IRS-1 Gly972Arg polymorphism in recessive genotype model.

Parameter	PCOS		*p* Value	Controls		*p* Value
	GG (*n* = 154)	GA + AA (*n* = 95)		GG (*n* = 56)	GA + AA (*n* = 44)	
Age (years)	22.21 ± 3.74	22.82 ± 4.72	0.25	22.11 ± 3.21	21.73 ± 3.00	0.58
Weight (kg)	59.12 ± 10.68	60.98 ± 12.91	0.24	50.77 ± 6.43	53.43 ± 6.86	0.04 *
Height (m)	1.58 ± 0.05	1.56 ± 0.05	0.05	1.56 ± 0.06	1.58 ± 0.05	0.07
BMI (kg/m^2^)	23.87 ± 4.41	24.96 ± 4.76	0.06	20.89 ± 2.32	21.52 ± 2.64	0.20
Waist (cm)	82.63 ± 10.88	83.86 ± 10.83	0.38	76.80 ± 7.41	77.27 ± 6.77	0.74
Hip (cm)	92.44 ± 8.25	95.15 ± 8.19	0.012 *	90.52 ± 6.26	92.30 ± 6.36	0.16
WHR	0.89 ± 0.09	0.88 ± 0.07	0.35	0.85 ± 0.06	0.84 ± 0.05	0.37
SBP (mmHg)	120.50 ± 7.36	120.67 ± 7.58	0.86	118.93 ± 5.54	119.00 ± 5.29	0.94
DBP (mmHg)	80.50 ± 5.46	81.07 ± 6.40	0.45	79.21 ± 5.27	79.45 ± 5.23	0.82
Menarche (years)	13.19 ± 1.14	13.05 ± 1.12	0.34	13.30 ± 1.04	13.25 ± 1.12	0.81
LH (IU/L)	12.28 ± 10.92	9.95 ± 6.62	0.06	6.51 ± 2.52	6.83 ± 2.14	0.50
FSH (IU/L)	5.94 ± 1.66	6.32 ± 2.15	0.11	6.87 ± 1.95	6.93 ± 2.03	0.88
TT (ng/dL)	61.22 ± 20.89	60.86 ± 26.20	0.90	33.90 ± 16.89	34.46 ± 13.97	0.85
PRL (ng/mL)	13.56 ± 5.84	12.86 ± 5.22	0.34	10.71 ± 5.28	10.06 ± 4.49	0.51
SHBG (nmol/L)	51.30 ± 23.33	48.10 ± 18.59	0.20	62.86 ± 23.34	67.51 ± 27.91	0.36
Andro (ng/mL)	3.32 ± 0.78	3.14 ± 1.03	0.11	2.30 ± 0.74	2.20 ± 0.62	0.47
Insulin F (μIU/mL)	12.86 ± 6.66	14.61 ± 7.22	0.05	7.45 ± 6.08	8.07 ± 4.41	0.57
Glu F (mg/dL)	85.37 ± 8.04	86.23 ± 9.43	0.44	84.31 ± 9.39	84.84 ± 8.26	0.76
Glu 2 h (mg/dL)	117.69 ± 19.11	112.82 ± 17.21	0.04 *	109.25 ± 15.87	107.66 ± 12.09	0.58
Chol (mg/dL)	157.56 ± 36.63	150.14 ± 30.78	0.10	132.57 ± 18.98	138.34 ± 19.44	0.13
TG (mg/dL)	121.13 ± 38.75	119.73 ± 29.88	0.76	101.20 ± 13.18	104.68 ± 16.54	0.24
QUICKI	0.331 ± 0.02	0.326 ± 0.02	0.05	0.38 ± 0.04	0.37 ± 0.05	0.26
FAI	5.89 ± 6.26	5.56 ± 4.40	0.65	2.20 ± 1.50	2.23 ± 1.86	0.92
LAP	34.38 ± 21.38	36.42 ± 22.55	0.49	21.64 ± 9.39	22.91 ± 8.74	0.49
Urea	23.45 ± 6.20	21.61 ± 5.43	0.01 *	21.38 ± 3.58	21.23 ± 3.37	0.83
Creatinine (mg/dL)	1.01 ± 0.44	1.06 ± 0.40	0.36	0.78 ± 0.12	0.83 ± 0.15	0.06
UA (mg/dL)	4.27 ± 1.06	4.28 ± 1.13	0.94	3.77 ± 0.66	3.94 ± 0.86	0.26
AST (U/L)	32.81 ± 13.78	29.10 ± 9.60	0.02 *	18.23 ± 7.83	18.40 ± 7.95	0.91
ALT (U/L)	29.08 ± 15.23	25.83 ± 10.95	0.07	23.01 ± 6.42	24.03 ± 7.25	0.45

Data presented as mean ± SD. * *p* value < 0.05 was considered significant as calculated by independent Student’s *t*-test. PCOS: polycystic ovary syndrome, BMI: body mass index, SBP: systolic blood pressure, DBP: diastolic blood pressure, FG score: Ferriman–Gallweyscore, LH: luteinizing hormone, FSH: follicle-stimulating hormone, TT: total testosterone, PRL: prolactin, TSH: thyroid-stimulating hormone, SHBG: sex hormone-binding globulin, Andro: androstenrdione, DHEAS: dihydroepiandrostenedione sulphate, Glu F: glucose fasting, CHOL: cholesterol, TG: triglycerides, QUICKI: quantitative insulin sensitivity check index, FAI: free androgen index, UA: uric acid, AST: aspartate aminotransferase, ALT: alanine aminotransferase.

**Table 5:** Genotypic–phenotypic association of clinical symptoms of hyperandrogenism, oligo-anovulation and insulin resistance in IRS-1 Gly972Arg genotypes in PCOS women.

Clinical Feature	GG (*n* = 154)	GA (*n* = 87)	AA (*n* = 8)	GA + AA (*n* = 95)	(χ^2^) *p* ^a^	(χ^2^) *p* ^b^
**Acne**	100 (64.93%)	38 (43.67%)	4 (50.00%)	42 (44.21%)	(10.41) 0.005	(10.29) 0.00
**Alopecia**	70 (45.45%)	24 (27.58%)	3 (37.5%)	27 (28.72%)	(7.47) 0.02	(7.17) 0.00
**Acanthosis**	46 (29.87%)	34 (39.08%)	3 (37.5%)	37 (39.36%)	(2.19) 0.33	(2.18) 0.13
**Hirsutism**	131 (85.06%)	56 (64.36%)	3 (37.5%)	58 (61.70%)	(20.06) <0.001	(18.52) 0.00
**Oligomenorrhea**	148 (96.1%)	57 (65.51%)	8 (100%)	65 (69.15%)	43.45) <0.001	(36.4) 0.00
**Obesity**	61 (39.61%)	45 (51.72%)	4 (50.00%)	49 (52.13%)	(3.42) 0.18	(3.41) 0.06
**Insulin resistance**	73 (47.40%)	46 (52.87%)	4 (50.00%)	50 (53.19%)	(0.67) 0.71	(0.64) 0.42

Data presented as number (percent). Clinical features defined as: hirsutism FGS ≥ 8, obesity BMI ≥ 25 kg/m^2^, insulin resistance HOMA ≥ 2.71, *p* value calculated by chi-squared test. (χ^2^) *p* ^a^ *p* value of GG vs. GA vs. AA calculated by chi-squared test, (χ^2^) *p* ^b^ *p* value of GG vs. GA + AA calculated by chi-squared test. Oligomenorrhea defined as <9 menstrual cycles per year.

**Table 6:** Comparison of the present study with previous studies of IRS-1 gene Gly972Arg polymorphism in PCOS.

Author	Year	Ethnicity/Population	Case/Controls	*p* Value
ElMkadem et al., 2001 [14]	2001	Caucasian/French	53/102	0.39
Sir-Petermann et al., 2004 [16]	2004	Caucasian/Chilean	143/97	0.10
Haap et al., 2005 [37]	2005	Caucasian/German	56/316	0.53
Villuendas et al., 2005 [34]	2005	Caucasian/Spanish	103/48	0.74
Dilek et al., 2005 [39]	2005	Caucasian/Turkish	60/60	0.03 *
Lin et al., 2006 [32]	2006	Asian/Taiwanese	47/45	NS
Valdés et al., 2008 [19]	2008	Caucasian/Chilean	50/75	0.18
Baba et al., 2007 [40]	2007	Asian/Japanese	123/380	0.014 *
Pappalardo et al., 2010 [41]	2010	Caucasian/Italian	65/27	<0.001 *
Skrgatic et al., 2013 [36]	2013	Caucasian/Croatian	150/170	0.25
Marioli et al., 2010 [29]	2010	Caucasian/Greek	162/122	0.81
Lin et al., 2014 [42]	2014	Asian/Taiwanese	248/92	0.50
Dasgupta et al., 2012 [30]	2012	South Asian/Indian	246/279	0.67
Oh et al., 2009 [31]	2009	Asian/Korean	125/344	>0.05
Shi et al., 2016 [21]	2016	Meta-analysis	1851/2017	0.004 *
Thangavelu et al., 2017 [18]	2017	South Asian/Indian	169/169	0.001 *
Pappalardo et al., 2016 [20]	2016	Caucasian/Italian	100/45	0.008 *
Christopoulos et al., 2010 [38]	2010	Caucasian/Greek	183/88	0.002 *
Present study	2022	South Asian/Kashmiri	249/100	0.49

* *p* Value of the study < 0.05.

## Data Availability

The data and materials will be provided by authors upon reasonable request.

## Supplementary Materials

The following supporting information can be downloaded at: https://www.mdpi.com/article/10.3390/genes13081463/s1, Table S1: Clinical characteristics, hormonal levels, metabolic and biochemical profile of IRS-1 SNP rs1801278 dominant genotype model in PCOS women, Table S2: Clinical characteristics, hormonal levels, metabolic and biochemical profile of IRS-1 Gly972 polymorphism in additive genotype model, Figure S1: Representative picture of PCR Amplification of IRS-1 gene containing rs1801278 SNP, Figure S2: Representative picture of digestion of 198 bp PCR product of IRS-1 SNP by SmaI.
