# Supplementary material for: Insulin Receptor Substrate 1 Gly972Arg (rs1801278) Polymorphism Is Associated with Obesity and Insulin Resistance in Kashmiri Women with Polycystic Ovary Syndrome
Source: Genes (Basel). 2022 Aug 17;13(8):1463. doi: 10.3390/genes13081463 (PMC9408134; doi:10.3390/genes13081463)
Supplement: Supplementary file 1 [file genes-13-01463-s001.zip › genes-1696296-supplementary.pdf]

Supplementary Material

Insulin Receptor Substrate 1 Gly972Arg (Rs1801278) Polymorphism Is Associated with Obesity and Insulin Resistance in Kashmiri Women with Polycystic Ovary Syndrome

Shayaq Ul Abeer Rasool [1], Sairish Ashraf [2], Mudasar Nabi [2], Shajrul Amin [2,*]

1. Department of Biotechnology, University of Kashmir, Srinagar India
2. Department of Biochemistry, University of Kashmir, Srinagar India

*Corresponding Author

Shajrul Amin
Professor & Head
Department of Biochemistry, University of Kashmir,
Srinagar-190006, India.
Tel: +91-9419018174
Email: shajrulamin@uok.edu.in

Table S1: Clinical characteristics, hormonal levels, metabolic and biochemical profile of RS-1 SNP rs1801278 dominant genotype model in PCOS women

Parameter	PCOS		P value	Controls		P Value
	GG+GA (n=241)	AA (n=8)		GG+GA (n=98)	AA (n=2)	
Age (years)	22.35 ±4.05	21.89 ±3.53	0.315	21.95 ±3.14	21.50 ±0.71	0.841
Weight (kg)	59.77 ±11.63	59.94 ±9.48	0.967	51.92 ±6.52	53.00 ±18.38	0.823
Height (m)	1.57 ±0.05	1.55 ±0.04	0.264	1.57 ±0.05	1.57 ±0.06	1.000
BMI (kg/m^2)	24.25 ±4.70	24.98 ±4.16	0.665	21.16 ±2.43	21.27 ±5.921	0.951
Waist (cm)	83.05 ±11.10	83.83 ±11.27	0.845	76.94 ±7.03	80.50 ±13.44	0.485
Hip (cm)	93.41 ±8.26	95.39 ±7.85	0.504	91.21 ±6.36	95.50 ±2.12	0.345
WHR	0.89 ±0.08	0.88 ±0.07	0.727	0.84 ±0.05	0.84 ±0.12	1.000
WHtR	0.53 ±0.07	0.54±0.07	0.691	0.49 ±0.04	0.51 ±0.07	0.490
BAI	29.54 ±4.69	31.44±4.32	0.259	28.58 ±3.05	30.58 ±1.55	0.359
SBP (mmHg)	120.46 ±7.48	117.94 ±7.36	0.349	119.06 ±5.34	114.00 ±8.49	0.191
DBP (mmHg)	80.65 ±5.87	80.91 ±6.56	0.902	79.39 ±5.23	76.00 ±5.66	0.367
Mena (years)	13.15 ±1.13	13.08 ±1.10	0.863	13.31 ±1.07	12.00 ±0.00	0.088
FG Score	14.03 ±6.49	12.72 ±6.90	0.575	4.51 ±1.84	5.50 ±2.12	0.453
LH (IU/L)	11.36 ±9.68	9.99 ±6.87	0.694	6.61 ±2.36	8.80 ±0.49	0.195
FSH (IU/L)	6.06 ±1.85	6.29 ±2.13	0.731	6.95 ±1.93	4.39 ±3.27	0.068
TT (ng/dL)	60.98 ±22.95	60.54 ±26.31	0.957	33.68 ±15.29	56.77 ±19.29	0.037*
PRL (ng/mL)	13.32 ±5.59	12.89 ±5.15	0.830	10.44 ±4.95	13.66 ±6.05	0.365
TSH (uIU/L)	3.21 ±1.45	3.40 ±1.39	0.715	3.00 ±1.48	2.63 ±1.05	0.726
SHBG (nmol/L)	50.18 ±21.65	48.19 ±18.28	0.797	65.21 ±25.42	50.17 ±29.40	0.410
Andro (ng/mL)	3.26 ±0.87	3.16 ±1.00	0.750	2.26 ±0.69	2.37 ±0.70	0.823
DHEAS (ng/mL)	3.79 ±1.15	3.56 ±1.15	0.578	2.92 ±1.31	2.38 ±0.78	0.569
Insulin F (μIU/ml)	13.54 ±6.86	14.74 ±7.09	0.627	7.64 ±5.35	11.80 ±8.25	0.282
Glu F (mg/dL)	85.64 ±8.54	86.11 ±9.38	0.878	84.43 ±8.88	90.00 ±8.49	0.381
Glu 2h (mg/dL)	115.70±18.21	112.17 ±15.98	0.588	108.41 ±14.36	115.50 ±10.61	0.49
Chol (mg/dL)	154.89 ±34.94	150.17 ±31.36	0.706	135.22 ±19.47	129.50 ±7.78	0.684
TG (mg/dL)	121.08 ±35.79	121.00 ±30.05	0.995	102.66 ±14.92	106.00 ±0.00	0.753
HOMA IR	2.90 ±1.62	3.19 ±1.71	0.619	1.59 ±1.16	2.71 ±2.08	0.184
QUICKI	0.33 ±0.02	0.33 ±0.02	1.000	0.38 ±0.05	0.34 ±0.04	0.264
FAI	5.74 ± 5.64	5.48 ±4.36	0.897	2.15 ±1.55	5.21 ±4.39	0.008*
LH:FSH	1.92 ±1.28	1.65± 0.97	0.555	1.00 ±0.45	2.72 ±1.92	0.001*
LAP	35.23 ±21.91	36.73±22.86	0.849	22.10 ±9.00	26.93 ±16.08	0.459
Urea	22.67 ±6.01	21.31 ±5.44	0.528	21.21 ±3.32	26.62 ±8.09	0.028*
Crea (mg/dL)	1.02 ±0.43	1.05 ±0.41	0.846	0.80 ±0.13	0.90 ±0.11	0.283
UA (mg/dL)	4.28 ±1.10	4.29 ±1.16	0.979	3.84 ±0.75	3.16 ±0.90	0.208
AST (U/L)	31. 29±12.39	28.60±8.89	0.543	18.39 ±7.90	13.75 ±0.35	0.410
ALT (U/L)	27.89 ±13.86	25.78 ±10.77	0.670	23.56 ±6.78	18.35 ±6.86	0.285

Data presented as Mean ±SD. *P-value <0.05 significant. Data presented as Mean ±SD. *P-value <0.05 significant. P values calculated by Student's t test

PCOS polycystic ovary syndrome, BMI body mass index, SBP systolic blood pressure, DBP diastolic blood pressure, FG Score ferriman gallwey score, LH luteinizing hormone, FSH follicle stimulating hormone, TT total testosterone, PRL Prolactin, TSH thyroid stimulating hormone, SHBG sex hormone binding globin, Andro androstenrdione,DHEAS dihydroepiandrostenedione sulphate, Glu F glucose fasting, CHOL cholesterol, TG triglycerides, HOMA IR homeostasis model assessment-estimated insulin resistance, QUICKI quantitative insulin sensitivity check index, FAI free androgen index, UA uric acid, AST aspartate aminotransferase, ALT alanine aminotransferase.

Table S2: Clinical characteristics, hormonal levels, metabolic and biochemical profile of IRS-1 Gly972 polymorphism in additive genotype model

Parameter	PCOS			P value	Controls			P Value
	GG (n=154)	GA (n=87)	AA (n=8)		GG (n=56)	GA (n=42)	AA (n=2)	
Age (years)	22.21 ±3.74	22.60 ±4.56	25.25 ±5.99	0.117	22.11 ±3.21	21.83 ±3.09	21.5 ±0.71	0.827
Weight (kg)	59.12 ±10.68	60.91 ±13.15	61.75 ±10.67	0.464	50.77 ±6.43	53.68 ±6.39	53.00 ±18.38	0.143
Height (m)	1.58 ±0.05	1.56 ±0.05	1.56 ±0.06	0.194	1.56 ±0.06	1.58 ±0.05	1.57 ±0.06	0.277
BMI (kg/m^2)	23.87 ±4.41	24.92±5.13	25.38 ±5.02	0.196	20.89 ±2.32	21.63 ±2.55	21.27 ±5.921	0.443
Waist (cm)	82.63 ±10.88	83.80 ±11.49	84.50 ±12.92	0.691	76.80 ±7.41	77.35 ±6.63	80.50 ±13.44	0.763
Hip (cm)	92.44 ±8.25	95.14 ±8.04	95.25 ±8.61	0.41	90.52 ±6.26	92.25 ±6.57	95.50 ±2.12	0.291
WHR	0.89 ±0.09	0.88 ±0.07	0.88 ±0.09	0.376	0.85 ±0.06	0.84 ±0.04	0.84 ±0.12	0.620
WHtR	0.52±0.07	0.54±0.07	0.54±0.09	0.437	0.49 ±0.05	0.49 ±0.04	0.51 ±0.07	0.748
BAI	28.86±4.84	30.74±4.17	30.89±5.92	0.002*	28.56 ±3.04	28.67 ±3.13	30.58 ±1.55	0.658
SBP (mmHg)	120.50 ±7.36	120.39±7.72	123.75 ±5.18	0.468	118.93 ±5.54	119.20 ±5.26	114.00 ±8.49	0.409
DBP (mmHg)	80.50 ±5.46	80.91 ±6.56	82.88 ±4.19	0.497	79.21 ±5.27	79.60 ±5.36	76.00 ±5.66	0.620
Mena (years)	13.19 ±1.14	13.08 ±1.10	12.75 ±1.39	0.464	13.30 ±1.04	13.28 ±1.13	12.00 ±0.00	0.237
FG Score	14.77 ±6.15	12.72 ±6.90	11.63 ±9.26	0.04	4.63 ±2.02	4.35 ±1.58	5.50 ±2.12	0.590
LH (IU/L)	12.28 ±10.92	9.99 ±6.87	9.48 ±3.03	0.212	6.51 ±2.52	6.63 ±2.15	8.80 ±0.49	0.386
FSH (IU/L)	5.94 ±1.66	6.29 ±2.13	6.75 ±2.43	0.225	6.87 ±1.95	6.95 ±1.92	4.39 ±3.27	0.174
TT (ng/dL)	61.22±20.89	60.54 ±26.31	64.73 ±26.44	0.895	33.90 ±16.89	33.40 ±13.35	56.77 ±19.29	0.115
PRL (ng/mL)	13.56 ±5.84	12.89 ±5.15	12.43 ±6.31	0.619	10.71 ±5.28	10.10 ±4.58	13.66 ±6.05	0.811
TSH (uIU/L)	3.10 ±1.48	3.40 ±1.39	2.99 ±1.12	0.076	3.05 ±1.73	2.91 ±1.08	2.63 ±1.05	0.878
SHBG (nmol/L)	51.30 ±23.33	48.19 ±18.28	47.08 ±23.09	0.522	62.86 ±23.34	67.69 ±27.75	50.17 ±29.40	0.409
Andro (ng/mL)	3.32 ±0.78	3.16 ±1.00	3.30 ±0.74	0.334	2.30 ±0.74	2.22 ±0.63	2.37 ±0.70	0.734
DHEAS(ng/mL)	3.92 ±1.13	3.56 ±1.15	3.75 ±1.30	0.066	2.98 ±1.46	2.82 ±1.05	2.38 ±0.78	0.734
Insulin(µIU/ml)	12.86 ±6.66	14.74 ±7.09	13.20 ±9.01	0.128	7.45 ±6.08	8.05 ±4.29	11.80 ±8.25	0.519
Glu F (mg/dL)	85.37 ±8.04	86.11 ±9.38	87.50 ±10.58	0.677	84.31 ±9.39	84.13 ±8.17	90.00 ±8.49	0.678
Glu 2h(mg/dL)	117.69 ±19.11	112.17 ±15.98	119.88 ±27.90	0.069	109.25 ±15.87	107.33 ±12.43	115.50 ±10.61	0.632
Chol (mg/dL)	157.56 ±36.63	150.17 ±31.36	149.75 ±25.28	0.261	132.57 ±18.98	137.83 ±18.38	129.50 ±7.78	0.269
TG (mg/dL)	121.13 ±38.75	121.00 ±30.05	105.88 ±25.67	0.492	101.20 ±13.18	105.19 ±17.15	106.00 ±0.00	0.504
HOMA IR	2.74 ±1.55	3.19 ±1.71	2.97 ±2.21	0.114	1.56 ±1.33	1.66 ±0.91	2.71 ±2.08	0.394
QUICKI	0.331 ±0.02	0.325 ±0.02	0.334 ±0.03	0.091	0.38 ±0.04	0.37 ±0.05	0.34 ±0.04	0.279
FAI	5.89 ±6.26	5.48 ±4.36	6.47 ±5.13	0.802	2.20 ±1.50	2.11 ±1.67	5.21 ±4.39	0.031
LH:FSH	2.07 ±1.41	1.65± 0.97	1.60 ±0.76	0.041	1.01 ±0.51[b]	0.99 ±0.37[c]	2.72 ±1.92	0.001
LAP	34.38±21.38	36.73±22.86	33.08±19.83	0.698	21.64 ±9.39	23.10 ±8.56	26.93 ±16.08	0.645
Urea (mg/dL)	23.45 ±6.20	21.31 ±5.44	24.88 ±4.39	0.016*	21.38 ±3.58	20.89 ±2.93	26.62 ±8.09	0.077
Crea (mg/dL)	1.01 ±0.44	1.05 ±0.41	1.19 ±0.39	0.459	0.78 ±0.12	0.83 ±0.15	0.90 ±0.11	0.127
UA (mg/dL)	4.27 ±1.06	4.29 ±1.16	4.13 ±0.79	0.932	3.77 ±0.66	3.95 ±0.88	3.16 ±0.90	0.524

AST (U/L)	32.81 ±13.78	28.60±8.89	34.63 ±15.10	0.031*	18.23 ±7.83	18.70 ±8.23	13.75 ±0.35	0.691
ALT (U/L)	29.08 ±15.23	25.78 ±10.77	26.38 ±13.55	0.198	23.01 ±6.42	24.56 ±7.32	18.35 ±6.86	0.368

Data presented as Mean ±SD. *P-value <0.05 significant. P values calculated by one way Analysis of Variance (ANOVA) independent standard weighted-means analysis and intergroup association tested by Post hoc Tukey HSD test.

PCOS polycystic ovary syndrome, BMI body mass index, SBP systolic blood pressure, DBP diastolic blood pressure, FG Score ferriman gallwey score, LH luteinizing hormone, FSH follicle stimulating hormone, TT total testosterone, PRL Prolactin, TSH thyroid stimulating hormone, SHBG sex hormone binding globin, Andro androstenrdione, DHEAS dihydroepiandrostenedione sulphate, Glu F glucose fasting, CHOL cholesterol, TG triglycerides, HOMA IR homeostasis model assessment-estimated insulin resistance, QUICKI quantitative insulin sensitivity check index, FAI free androgen index, UA uric acid, AST aspartate aminotransferase, ALT alanine aminotransferase.

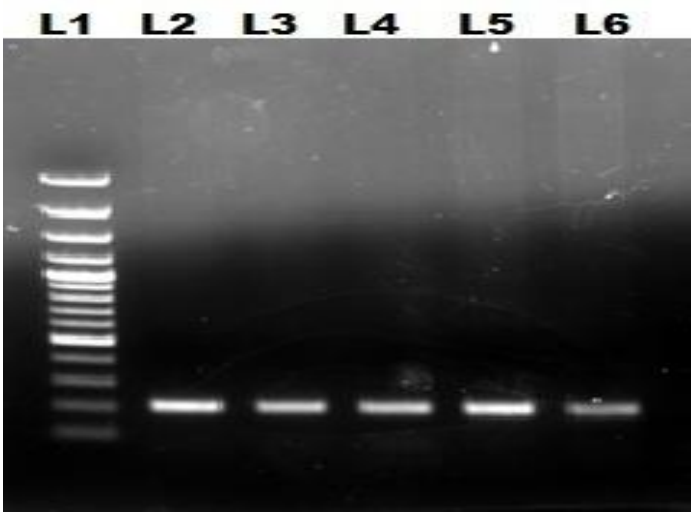

Figure S1: Representative picture of PCR Amplification of IRS-1 gene containg rs1801278 SNP (Lane 1 shows 100bp DNA ladder, Lanes 2-6 show 198bp PCR product)

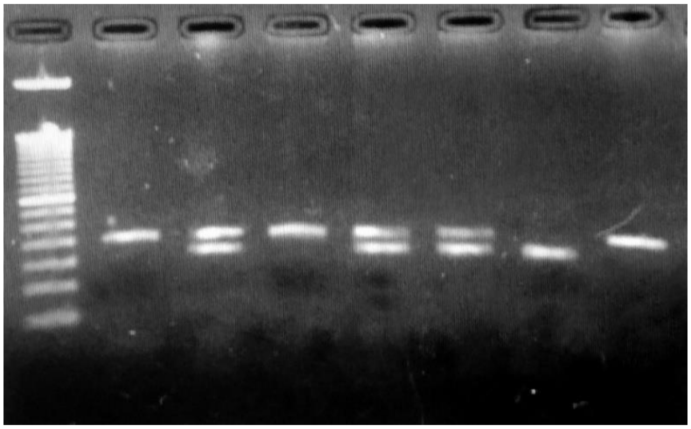

Figure S2: Representative picture of digestion of 198 bp PCR product of IRS-1 SNP by SmaI

Lane 1 shows 50bp Ladder, Lanes 2,4 and 8 show wild GG genotype represented by undigested 198bp PCR product, lanes 3, 5 and 6 show heterozygous GA genotype represented by 198bp+171bp fragments and lane 7 shows AA genotype represented by 171bpbp fragments
